# Bone Density and Hyperlipidemia: The T-lymphocyte Connection

**DOI:** 10.1002/jbmr.148

**Published:** 2010-06-07

**Authors:** Lucia S Graham, Yin Tintut, Farhad Parhami, Christina MR Kitchen, Yevgeniv Ivanov, Sotirios Tetradis, Rita B Effros

**Affiliations:** 1 Department of Pathology and Laboratory Medicine and David Geffen School of Medicine at the University of California Los Angeles Los Angeles, CA, USA; 2 Department of Medicine, David Geffen School of Medicine at the University of California Los Angeles Los Angeles, CA, USA; 3 Department of Biostatistics, University of California Los Angeles School of Public Health Los Angeles, CA, USA; 4 Division of Diagnostic and Surgical Sciences, University of California Los Angeles School of Dentistry Los Angeles, CA, USA

**Keywords:** T-lymphocytes, lipids, osteoporosis, cytokines, inflammation

## Abstract

Osteoporosis, which contributes to morbidity and mortality, often coexists with cardiovascular disease, especially atherosclerosis. We have reported recently that in vitro exposure of human T-lymphocytes to oxidized lipids induced expression of a key osteoclastogenic cytokine, receptor activator of NF-κB ligand (RANKL). Our previous studies have shown that mice fed an atherogenic high-fat diet developed osteopenia and that bone marrow preosteoclasts from these hyperlipidemic mice have increased osteoclastic potential. To investigate the role of T-lymphocytes in the diet-induced bone loss, C57BL/6 mice were fed either chow or a high-fat diet, and bone parameters and T-lymphocyte activation were assessed at 6 and 11 months. Consistent with our previous findings, peripheral quantitative computed tomographic (pQCT) analysis showed that mice in the high-fat group had lower bone mineral content than mice in the chow group. Furthermore, histomorphometric analysis showed decreased structural parameters in the high-fat group. Coculture studies showed that bone marrow cells isolated from the high-fat group, which contained increased levels of activated memory T-lymphocytes compared with bone marrow cells from the chow mice, supported osteoclastic differentiation of RAW 264.7 cells. Additionally, RANKL expression was upregulated significantly in the T-lymphocytes isolated from the bone marrow of the high-fat group. Splenic T-lymphocytes isolated from the high-fat group also had increased expression of transcripts for the receptor for oxidized lipids (LOX-1) as well as for inflammatory and osteoclastogenic cytokines, including RANKL, interleukin 6 (IL-6), tumor necrosis factor α (TNF-α), IL-1β, and interferon γ (IFN-γ). Together these findings suggest that T-lymphocytes play a key role in the osteoclastogenesis induced by a high-fat diet and may contribute to the bone loss associated with diet-induced osteopenia. © 2010 American Society for Bone and Mineral Research.

## Introduction

The pathophysiology of osteoporosis is complex and multifactorial, particularly with respect to the modulatory effects of the immune system on bone homeostasis. A central component of the interaction between the immune and skeletal systems is the tumor necrosis factor (TNF) receptor family molecule receptor activator of NF-κB ligand (RANKL) produced by bone marrow stromal cells,(1) osteoblasts,(2) and activated T-lymphocytes.(3) Indeed, activated T-lymphocytes have been shown to directly induce osteoclast formation from monocytes in vitro.(4) Furthermore, T-lymphocyte-deficient mice have decreased alveolar bone loss induced by oral infection compared with their immunocompetent counterparts.(5) Elevated serum levels of inflammatory cytokines such as interleukin 6 (IL-6), TNF-α, IL-1β, and interferon γ (IFN-γ) have been documented in diseases that are also associated with bone loss, including rheumatoid arthritis (RA),(6) inflammatory bowel disease (IBD),(7) and periodontal disease.(8)

Hyperlipidemia increases the risk of retention and modification of low-density lipoprotein (LDL) in arterial walls, which can undergo oxidation in macrophage-rich tissues.(9) Oxidized LDL is immunogenic and can recruit immune cells, including monocytes and T-lymphocytes, to the subendothelial layer of the artery wall.(10,11) Indeed, T-lymphocytes have been shown to be present at all stages of lesion development in atherosclerosis.(10) A more definitive demonstration of T-lymphocyte involvement in atherosclerosis emerged from studies of severely combined immunodeficient (SCID) mice that were crossed with *apoE*^−/−^ mice, where the reduction in fatty streak aorta lesions was reversed when the mice were reconstituted with T-lymphocytes.(12) Additionally, immune responses to oxidized lipids also have been documented in regional lymph nodes and spleen.(13,14) The primary receptor for oxidized LDL, lectin-like oxidized LDL receptor 1 (LOX-1), which has been associated with cardiovascular disease,(15) has been identified on endothelial cells.(16) In the Jurkat T-lymphocyte tumor line, *LOX1* mRNA was upregulated by lysophosphatidylcholine, a lipid component of oxidized LDL.(17) However, its expression in normal T-lymphocytes has not been investigated.

Cardiovascular disease, especially atherosclerosis, coexists in patients with osteoporosis.(18) We and others have reported the accumulation of lipids within the bones of mice and blood vessels of patients with osteoporosis.(19,20) In addition, we have found previously that mice placed on a high-fat diet developed not only atherosclerosis but also osteopenia.(21) We also showed that oxidized lipids enhance osteoblastic differentiation of bone marrow preosteoclasts.(22) Consistent with these findings, bone marrow preosteoclasts isolated from hyperlipidemic mice have greater osteoclastic potential than similar cells from normolipemic mice.(20) Therefore, the high-fat diet not only may inhibit differentiation of osteoblasts but also may promote differentiation of osteoclasts for bone resorption.

An often neglected observation is that fully functional T-lymphocytes migrate back and forth between blood and bone marrow (BM).(23,24) It seems likely, therefore, that the loosely compartmentalized space within the BM may allow immune and bone cells to interact with and to influence each other. Interestingly, memory CD8 T-lymphocytes isolated from the BM are in a more activated state and are more abundant than similar cells in the periphery.(25) The increased proportion of CD8 T-lymphocytes within the BM, in fact, may account for the inverted CD4/CD8 ratio (1:2) in that tissue compared with the 2:1 ratio in the periphery.(26) Importantly, following antigenic activation, memory, but not naive, T-lymphocytes express RANKL, and these RANKL-expressing memory cells preferentially reside in the bone.(27)

Inflammatory processes are known to be associated with decreased bone mass, but the specific involvement of T-lymphocytes in hyperlipidemia-induced bone loss has not been adequately addressed. Since activated T-lymphocytes produce cytokines that affect the maturation and differentiation of osteoclasts, in this study we used an in vivo model to investigate the contribution of T-lymphocytes to lipid-mediated bone loss. Our results show that splenic T-lymphocytes isolated from mice fed a high-fat diet had increased transcripts for multiple osteoclastogenic cytokines and for the LOX-1 oxidized lipid receptor. In addition, bone marrow from the high-fat group contained higher proportions of activated memory T-lymphocytes and supported osteoclast differentiation when cocultured with RAW 264.7 cells. These findings suggest that T-lymphocytes involved in the inflammatory response of atherosclerosis also may be mediators of lipid-induced bone loss and may constitute previously unrecognized common denominators in the pathogenesis of osteoporosis and atherosclerosis.

## Materials and Methods

### Mice and diets

Male C57Bl/6 mice (Jackson Laboratory, Bar Harbor, ME, USA) at 1 month of age were placed on either a standard chow diet (National Institute of Health 31 Mouse/Rat Diet 7013 containing 6% fat; NIH, Bethesda, MD, USA) or a high-fat (atherogenic) diet (Teklad TD90221, containing 1.25% cholesterol, 15.8% fat, and 0.5% cholate; Harland Teklad, Madison, WI, USA). In this mouse strain, the high-fat diet causes significant hypercholesterolemia(28,29) and osteopenia.(21) Femur, tibia, spleen, and plasma were collected at 6 (*n* = 10/group) and 11 (*n* = 4/group) months of age. The experimental protocol was reviewed and approved by the Institutional Animal Care and Use Committee of the University of California Los Angeles. Lipid analysis was performed by the University of California Los Angeles (UCLA) Atherosclerosis Research Unit core laboratory, and c-LDL was determined using Friedwald's formula(30):


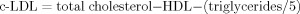


### Isolation of T-lymphocytes and bone marrow cells

T-lymphocytes were isolated from the total splenocyte population by immunomagnetic depletion using a mixture of biotinylated mAbs to CD14, CD16, CD19, CD56, CD36, CD123, and CD235a and subsequently labeled with antibiotin microbeads (Miltenyi Biotec, Auburn, CA, USA). Purity of T-lymphocytes (>98%) was confirmed by flow cytometry (FACS Calibur, Becton Dickinson, Franklin Lakes, NJ, USA) using anti-CD3 antibody (BD Pharmingen, San Diego, CA, USA). Bone marrow cells were flushed out with α-MEM from the femurs and tibias using 18-gauge needles and passed through a 40 µM nylon mesh cell strainer to make a single-cell suspension. For some coculture assays, whole bone marrow cells were incubated with biotinylated CD3ɛ^+^ monoclonal antibody, labeled with antibiotin microbeads, and loaded onto a MACS column (Miltenyi Biotec). The flow-through (ie, bone marrow cells depleted of T-lymphocytes) was washed with α-MEM and used immediately in the coculture assay.

### Quantitative computed tomographic scanning

Femurs and tibias were carefully cleaned of soft tissue and fixed in 95% ethanol. Peripheral quantitative computed tomographic (pQCT) scans were performed on the left femur and tibia from each mouse at 6 and 11 months. Scanning was performed with a Stratec XCT-Research M (Norland Medical Systems, Fort Atkinson, WI, USA) and associated software (Version 5.4, Stratec Medizintechnik, Pforzheim, Germany), and bone mineral content (BMC) analysis was performed as described previously.(31) Based on image morphology, the mean of six slices located in the midshaft of the femur and tibia was selected for data analysis.

### Micro–computed tomographic (µCT) analysis

µCT analysis was performed on the left femur and tibia from each mouse at 11 months. A high-resolution µCT scanner (mCT40, Scanco, Akron, OH, USA) was used.(30) µCT data were collected at 55 kVp and 72 mA at a resolution of 16 mm. Visualization, reconstruction, and volume analysis of the data were performed using the MetaMorph*1*Imaging System (Universal Imaging Corporation, Downingtown, PA, USA). Bone-specific analyses included new bone volume (BV), tissue volume (TV), and the BV/TV ratio.

### Trabecular bone histomorphometric analysis

Left femurs from each mouse at 11 months were subjected to static histomorphometry. Bones were fixed in 95% ethanol, dehydrated, and embedded undecalcified in methyl methacrylate. Longitudinal sections 5 µm thick were cut on a Microm microtome (Microm, Richards-Allan Scientific, Kalamazoo, MI, USA) and stained with toluidine blue (pH 6.4). Static parameters of bone formation and resorption were measured in a defined area between 181 and 725 µm from the growth plate using an OsteoMeasure morphometry system (Osteometrics, Atlanta, GA, USA). The terminology and units used are those recommended by the Histomorphometry Nomenclature Committee of the American Society for Bone and Mineral Research.

### Cytokine measurement

Plasma levels of RANKL, osteoprotegerin (OPG), IL-6, and IFN-γ were measured in triplicate wells by Quantikine ELISA (R&D Systems, Minneapolis, MN, USA) in accordance with manufacturer's instructions.

### Quantitative real-time PCR (RT-qPCR)

Total RNA from purified splenic and bone marrow T-lymphocytes was extracted using the RNeasy Mini Kit (Qiagen, Valencia, CA, USA), and RNA concentrations were determined using the Quant-iT Ribogreen RNA Assay Kit (Molecular Probes, Eugene, OR, USA). Template cDNAs were synthesized with the iScript cDNA Synthesis Kit (Bio-Rad, Hercules, CA, USA) using 500 ng of RNA. The QT-PCR assays were performed using the iQ SYBR Green SuperMix and IQCycler (Bio-Rad). *GAPDH* was used as a normalization control. The sequences of the primers were designed with the aid of Beacon Designer™ Software (Palo Alto, CA, USA). Primer sequences for each gene are listed in Table 1. Samples were run in triplicate in a 96-well plate using the settings of 95°C for 15 seconds, 61 to 60°C for 30 seconds, and 72°C for 30 seconds (single fluorescence measurement).

**Table 1:** Primer Sequences Used for Quantitative Real-Time Polymerase Chain Reaction

Gene	Primer sequence	Ta
IFN-γ	F - CAAGTGGCATAGATGTGGAAGAAAAGAG R - AATGACGCTTATGTTGTTGCTGATGG	61
IL1-β	F - GCAGCAGCACATCAACAAGAGC R - CCACGGGAAAGACACAGGTAGC	61
IL-6	F - CCTTCTTGGGACTGATGCTGGTG R-TGGTATCCTCTGTGAAGTCTCCTCTC	61
LOX-1	F - CCTCTACCTCAGTATGCCTCCT R - CTTTGCATGTTATTTCTCGGACGA	60
OPG	F - CCTTGCCCTGACCACTCTTATACG R-CCTTCCTCACACTCACACACTCG	61
RANKL	F - GCCTCCCGCTCCATGTTCC R - TGAGTGCTGTCTTCTGATATTCTGTTAG	61
TNF-α	F - GGAACTGGCAGAAGAGGCACTC R - GAATGAGAAGAGGCTGAGACATAGGC	61
GAPDH	F - ATTGTCAGCAATGCATCCTG R-ATGGACTGTGGTCATGAGCC	60

Ta: annealing temperature.

### Flow cytometry

Phenotypic analysis of bone marrow cells was assessed by flow cytometry. Briefly, cells were washed twice with PBS and resuspended in staining buffer (BD Biosciences, San Jose, CA, USA). Cells were incubated with CD3 FITC, CD8 PE, CD4 PerCp, CD44 APC, CD62L PE, and CD69 PerCp (BD Biosciences) for 20 minutes at 4°C in the dark, washed, and resuspended in 1% paraformaldehyde. Data were acquired by BD FACS Calibur (BD Biosciences) and analyzed with CellQuest Pro (BD Biosciences).

### Osteoclast assays

The ability of bone marrow cells to support osteoclast formation was determined by coculturing bone marrow cells (0.3 × 10^6^ cells/well) with the RAW 264.7 mouse monocytic cell line (ATCC, Manassas, VA, USA) at 0.5 × 10^3^ cells/well in 96-well plates for 10 days. Cells were cultured in α-MEM supplemented with 10% fetal bovine serum (FBS), 50 IU/mL of penicillin/streptomycin, 1% sodium pyruvate, and 20 IU/mL of recombinant human IL-2. The medium was replaced every 3 days. At the end of the culture period, tartrate-resistant acid phosphatase (TRAP) staining was performed using a commercial kit (Sigma, St Loius, MO, USA). Multinucleated (>3 nuclei) TRAP^+^ cells were identified as osteoclasts and enumerated by light microscopy.

### Statistical analysis

Data in the graphs indicate the mean ± SD calculated from independent experiments unless otherwise specified. The Wilcoxon rank-sum test, a nonparametric test that is powerful in analyzing small sample sizes,(32) was used to compare groups. Statistical significance was defined as *p* ≤ .05.

## Results

### Effect of high-fat diet on plasma lipid levels and bone parameters

**Fig. 1:**
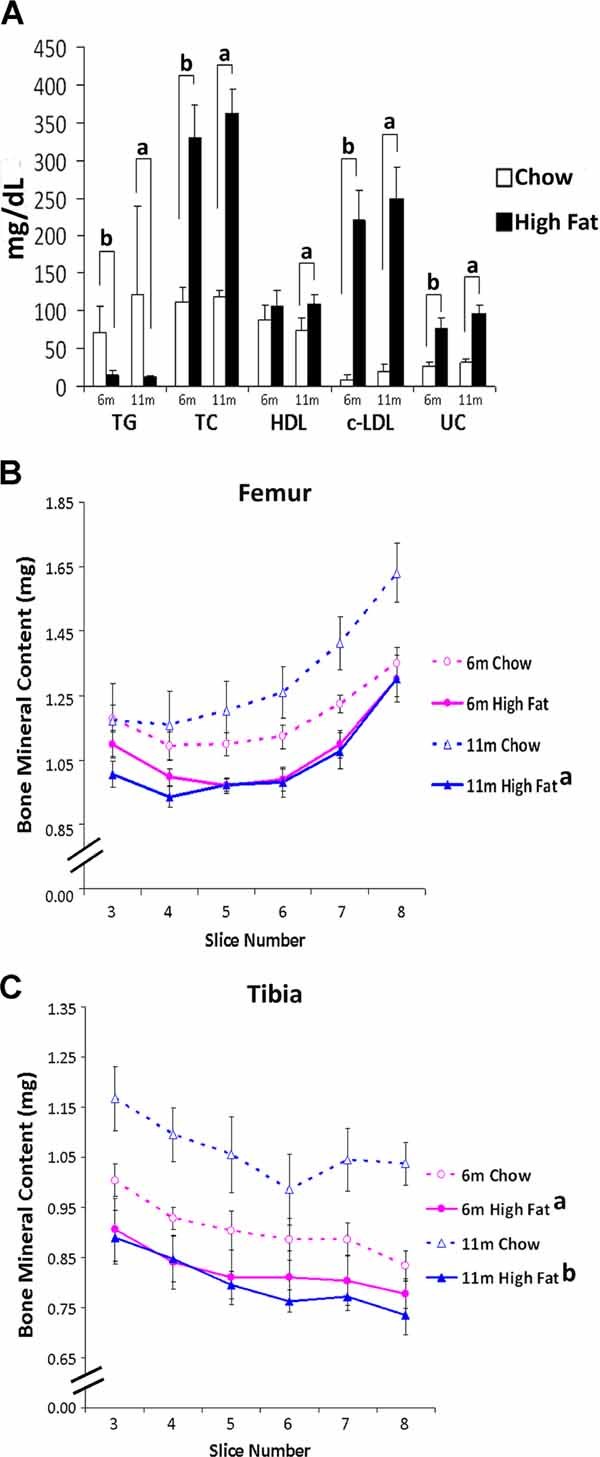
Plasma lipoprotein levels and pQCT analysis of femoral tibial bone. (*A*) Plasma triglycerides (TGs), total cholesterol (TC), HDL, c-LDL, and unesterified cholesterol (UC) of chow and high-fat groups. Error bars indicate SD. (*B*) Total BMC of femur was determined by pQCT. (*C*) Total BMC of tibia was determined by pQCT. Data are expressed as mean ± SE. ^a^*p* ≤ .05; ^b^*p* ≤ .01 compared to chow-fed control.

Hyperlipidemia was induced in C57Bl/6 mice that were fed a high-fat diet starting at 1 month of age, consistent with previous reports.(21) The diet significantly increased total cholesterol (TC), low-density lipoprotein (c-LDL), and unesterified cholesterol (UC) in the high-fat group compared with the chow group based on analyses of 6- and 11-month-old mice (Fig. 1*A*). pQCT analysis showed that while femoral and tibial bone mineral content (BMC) increased from 6 to 11 months in the chow group, as expected with growing mice, BMC of mice in the high-fat group did not increase (Figs. 1*B*, *C*). Although the body weight increased from 6 to 11 months in both groups, there was no significant difference between the chow and high-fat groups (data not shown). In addition, consistent with our previous findings, the BMC values of both femur and tibia were significantly lower in the high-fat group than in the chow group by 9% and 21–25% at 6 and 11 months, respectively (Fig. 1*B, C*). Bone mineral density (BMD) values of the femur and tibia also showed a nonsignificant trend toward decrease in the high-fat group at both 6 and 11 months (data not shown). Futhermore, histomorphometric analysis of femoral bone at 11 months showed a significant decrease in structural parameters in the high-fat group, including trabecular bone volume (BV/TV, 42.7%), trabecular thickness (Tb.Th, 30.7%), and trabecular number (Tb.N, 20%) with an increase in trabecular separation (Tb.Sp, 30.5%; Table 2). µCT analysis of left femurs and tibias is presented in Table 3. The high-fat group had significantly reduced bone structure in both femur and tibia, including cross-sectional tissue area (T.Ar), cross-sectional tissue perimeter (T.Pm), and cross-sectional bone area (B.Ar). Tibial bone from the high-fat group also had significantly lower cross-sectional bone perimeter (B.Pm), cross-sectional thickness (Cs.Th), cortical thickness, and cortical bone area (B.Ar/T.Ar, 8.6%) and the femoral bone showing similar decreases with a trend toward significance (Table 3).

**Table 2:** Histomorphometric Analysis of Trabecular Bone From Femurs of C57BI/6 Mice After 11 Months on Chow or High Fat Diet

	Chow	High Fat	Percent change
TV (mm)	0.63 ± 0.02	0.58 ± 0.04	−8.8
BV (mm)	0.09 ± 0.01	0.05 ± 0.01^b^	−47.2
BV/TV (%)	13.61 ± 2.09	7.8 ± 1.72^b^	−42.7
Tb.Th (mm)	29.57 ± 4.27	20.51 ± 3.20^a^	−30.7
Tb.Sp (mm)	196.93 ± 26.42	256.96 ± 24.28^a^	+30.5
Tb.N (/mm)	4.62 ± 0.51	3.70 ± 0.33^a^	−20.0

TV: Tissue Volume; BV: Bone Volume; Tb.Th: Trabecular Thickness; Tb. Sp: Trabecular Separation; Tb.N.: Trabecular Number Data represented as mean ± SE.

a *p* ≤ 0.05

b *p* ≤ 0.01 compared to chow-fed control.

**Table 3:** MicroCT Analysis of Femoral and Tibial Bones From C57BI/6 Mice After 11 Months on Chow or High Fat Diet

	Femur			Tiba		
		
	Chow	High Fat	% change	Chow	High Fat	% change
T.Ar (mm^2^)	2.207 ± 0.012	1.954 ± 0.060^b^	−11.5	1.340 ± 0.012	1.139 ± 0.042^b^	−15.1
T.Pm (mm)	5.801 ± 0.027	5.482 ± 0.089^a^	−5.5	4.562 ± 0.037	4.194 ± 0.078^b^	−8.1
B.Ar (mm^2^)	0.877 ± 0.054	0.700 ± 0.033^a^	−20.3	0.686 ± 0.028	0.532 ± 0.022^b^	−22.4
B.Pm (mm)	10.425 ± 0.056	10.002 ± 0.167†	−4.1	7.866 ± 0.076	7.254 ± 0.130^b^	−7.8
Cs.Th (mm)	0.169 ± 0.011	0.140 ± 0.006‡	−17.1	0.174 ± 0.008	0.147 ± 0.004^a^	−16.0
Cortical Th (mm)	0.021 ± 0.000	0.020 ± 0.000	−2.4	0.021 ± 0.000	0.020 ± 0.000^a^	−2.2
B.Ar/T.Ar (%)	0.398 ± 0.025	0.358 ± 0.011	−10.1	0.511 ± 0.018	0.467 ± 0.003^a^	−8.6

T.Ar: cross sectional Tissue Area; T.Pm: cross sectional Tissue Perimeter; B.Ar: cross sectional Bone Area; B.Pm: cross sectional Bone Perimeter; Cs.Th: Cross sectional Thickness Data represented as mean ± SE.

a *p* ≤ 0.05

b *p* ≤ 0.01

† *p* = 0.054

‡ *p* = 0.06 compared to chow-fed control.

### Effect of high-fat diet on splenic T-lymphocyte expression of inflammatory and osteoclastogenic cytokines

**Fig. 2:**
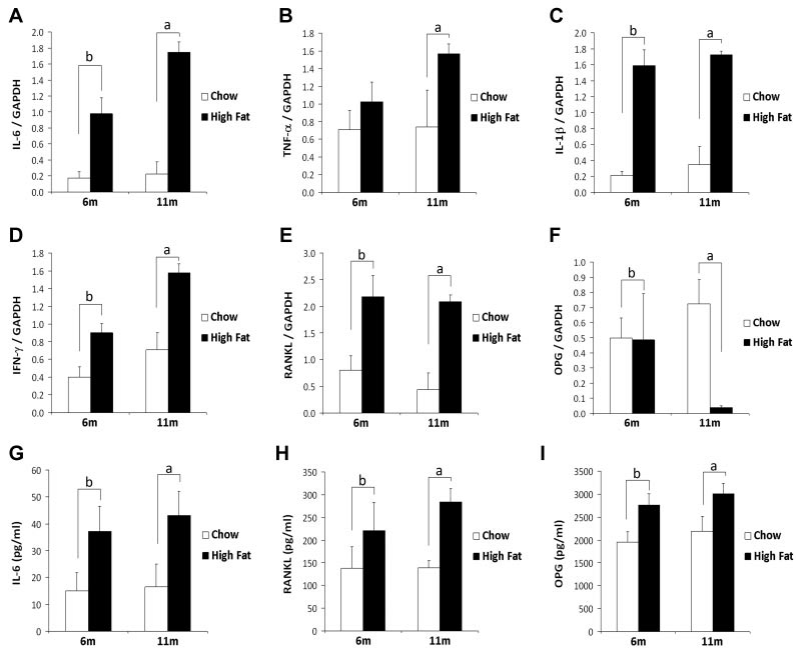
Message levels of inflammatory cytokines and osteogenic soluble factors. RT-qPCR analysis of gene expression compared with *GAPDH*. Relative expression of (*A*) IL-6, (*B*) TNF-α, (*C*) IL-1β, (*D*) IFN-γ, (*E*) RANKL, and (*F*) OPG by T-lymphocytes from either the chow or high-fat group. ELISA measurement of plasma (*G*) IL-6, (*H*) RANKL, and (*I*) OPG protein levels from high-fat and chow groups. Data are expressed as mean ± SD. ^a^*p* ≤ .05; ^b^*p* ≤ .01 compared to chow-fed control.

Hyperlipidemia has been documented extensively to cause a chronic inflammatory state by inducing the production of inflammatory cytokines,(33–35) including the cytokines known to induce bone resorption.(36,37) To determine the potential contribution of T-lymphocytes, one possible source of many of these inflammatory cytokines, we isolated splenic T-lymphocytes from both groups of mice and tested the message expression immediately ex vivo using RT-qPCR. Our analysis showed that the T-lymphocytes from the high-fat group had increased transcript levels for IL-6, TNF-α, IL-1β, IFN-γ, and RANKL and a decrease in OPG transcript expression (Fig. 2*A–F*). After measuring lipid levels in the plasma, we used the remaining plasma to evaluate circulating levels of several key osteoclastogenic cytokines. Consistent with the increased T-lymphocyte message levels, the plasma contained a corresponding increase in both IL-6 and RANKL proteins in the high-fat group at both 6 and 11 months (Fig. 2*G, H*). Circulating levels of IFN-γ also were increased at 6 months (data not shown) but, owing to the limited amount of plasma, could not be evaluated at 11 months. Interestingly, in contrast to the decreased OPG message in the T-lymphocytes, serum OPG levels actually were increased by the high-fat diet, suggesting that the circulating OPG may be derived from other sources (Fig. 2*I*). Nevertheless, the RANKL/OPG ratio, a key modulator of bone homeostasis, remained elevated in the high-fat diet mice at 11 months (0.047 for the chow group and 0.094 for the high-fat group), consistent with the observed bone loss seen in these mice.

### Effect of high-fat diet on T-lymphocyte LOX-1 expression

**Fig. 3:**
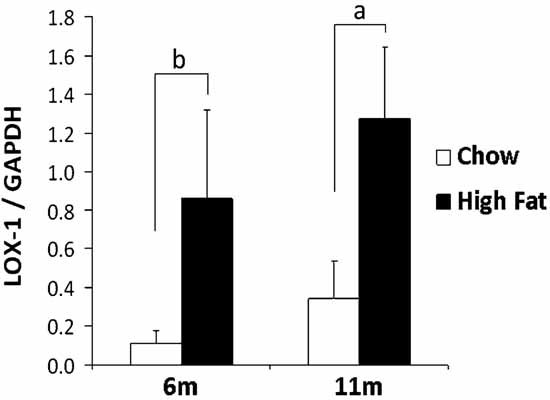
LOX-1 expression by T-lymphocytes. Relative gene expression (compared with *GAPDH*) of LOX-1 transcripts in splenic T-lymphocytes of chow and high-fat groups. Data are expressed as mean ± SD. ^a^*p* ≤ .05; ^b^*p* ≤ .01.

Since LOX-1 has been identified as the primary receptor for oxidized lipids in several non-immune cell types, we assessed its message expression in freshly isolated splenic T-lymphocytes. Results from RT-qPCR showed that LOX-1 message was increased in the high-fat group compared with the chow group at both 6 and 11 months (Fig. 3). This finding is consistent with our previous findings showing that in vitro exposure to oxidized lipids induced expression of LOX-1 in human T-lymphocytes.(38)

### Effect of high-fat diet on T-lymphocyte activation in bone marrow

**Fig. 4:**
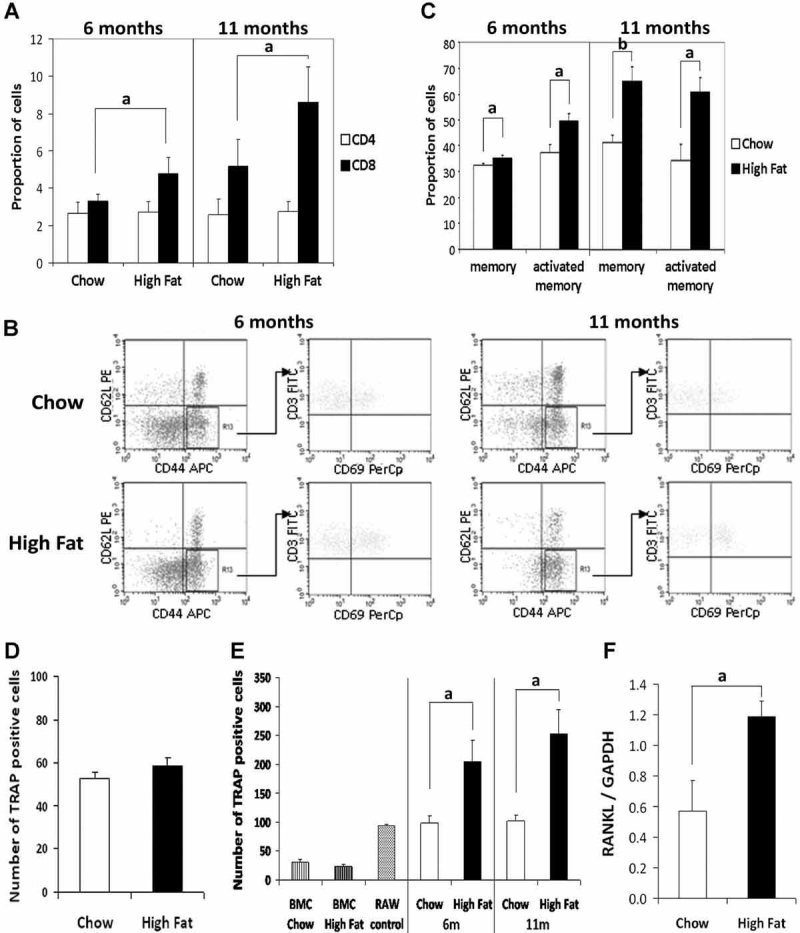
Phenotypic and functional analysis of bone marrow cells. Bone marrow cells isolated from the femur and tibia were analyzed phenotypically by flow cytometry and functionally based on their ability to support osteoclast maturation. (*A*) Mean proportion of CD44^hi^/CD4^+^ and CD44^hi^/CD8^+^ cells from BM of chow and high-fat mice at 6 and 11 months. (*B*) Representative flow cytometry dot plot; BM cells were first gated on T-lymphocytes (CD3^+^), and then the proportion of memory cells (CD44^hi^/CD62L^null^) was further analyzed for cell surface expression of the activation marker CD69. Representative dot-plot analysis of memory and activated memory T-lymphocytes from BM of chow and high-fat fed groups at 6 and 11 months. (*C*) Mean proportion of memory and activated memory T-lymphocytes in BM from chow and high-fat groups at 6 and 11 months. (*D*) Number of TRAP^+^ cells in cocultures containing T-lymphocyte-depleted BM cells from 6-month chow and high-fat groups. Each BM population was cocultured with RAW 264.7 cells for 10 days in the presence of 20 IU/mL of rh-IL-2. Data are expressed as mean ± SE. (*E*). BM cells from 6- and 11-month chow and high-fat groups were each cocultured with RAW 264.7 cells for 10 days in the presence of 20 IU/mL of rh-IL-2. Graph represents mean numbers of TRAP^+^ cells from cultures of chow BM alone, high-fat BM alone, RAW control, and RAW with BM cells from chow and high-fat groups. Data are expressed as mean ± SD. (*F*) RT-qPCR analysis of RANKL expression from T-lymphocytes isolated from BM cells from 6-month chow and high-fat groups. Data are expressed as mean ± SE. ^a^*p* ≤ .05; ^b^*p* ≤ .01.

Consistent with previous reports,(26) the relative proportion of CD8 (versus CD4) T-lymphocytes was increased in the BM of all the mice. However, the proportion of CD8 T-lymphocytes within the BM of the high-fat group was significantly higher than that in the chow group at both 6 and 11 months (Fig. 4*A*). The well-documented increase in memory and activated T-lymphocytes in the BM(39,40) also was exaggerated in the high-fat group. The representative flow cytometry dot-plot analysis (Fig. 4*B*) and the composite data (Fig. 4*C*) show that at both 6 and 11 months the BM of the high-fat group had a significantly higher proportion of memory T-lymphocytes, defined as CD3^+^CD44^hi^CD62L^null^ compared with the chow group. Moreover, within the total memory T-lymphocyte population, the proportion of cells that were in an activated state (ie, CD69^+^) was significantly higher in the high-fat group (Fig. 4*C*).

Since BM from the high-fat group contained increased level of activated T-lymphocytes, we sought to determine whether the ability of BM cells to support osteoclastic differentiation might be enhanced. BM cells were cocultured with the murine RAW 264.7 monocytic cell line for 10 days, and TRAP staining was performed to evaluate osteoclast numbers. The cultures were supplemented with IL-2 to ensure continued T-lymphocyte viability. Our data show that in the cocultures containing BM cells immunomagnetically depleted of T-lymphocytes, there were no significant difference in the number of multinucleated TRAP^+^ cells (Fig. 4*D*). However, the cocultures containing the whole BM (ie, with T-lymphocytes) from the high-fat group had significantly more TRAP^+^ osteoclasts than those from the chow group at both 6 and 11 months (Fig. 4*E*). Additionally, RANKL expression was increased in the isolated BM T-lymphocytes of the high-fat group compared with the chow group (Fig. 4*F*). The RAW 264.7 cells that were cultured in the absence of BM cells showed low but measurable numbers of TRAP^+^ cells, consistent with reports showing that IL-2 stimulates TRAP production in mouse calvarial bone.(41) Importantly, BM cell cultures that contained no RAW cells had a minimal number of TRAP^+^ cells, with a mean of fewer than 31 TRAP^+^ cells for chow and high-fat groups, confirming that the osteoclasts seen in the cocultures were derived from RAW cells (Fig. 4*E*). Although it is not possible to rule out a possible contribution of BM stromal osteoblast cells, our data suggest that the increased activated memory T-lymphocytes in the BM of the high-fat diet mice (Fig. 4*C*) may be responsible for the increased osteoclastic differentiation.

## Discussion

The role of lipid and lipoprotein oxidation in the pathophysiology of osteoporosis has been suggested by a variety of studies.(42,43) Consistent with our previous report, mice that were fed an atherogenic high-fat diet not only became hyperlipidemic but also showed significantly reduced mineral content and BV/TV in both the femoral and tibial bones. The major new findings of this study relate to the key role of T-lymphocyte in hyperlipidemia-induced bone loss in these mice. Specifically, we demonstrate that T-lymphocytes isolated from the spleen and BM from the high-fat group showed increased expression of RANKL message. Moreover, splenic T-lymphocytes had reduced OPG message expression. Increased RANKL expression also was seen at the protein level within the plasma of these mice. Additionally, T-lymphocytes from the high-fat group expressed transcripts for several other osteoclastogenic cytokines. Furthermore, the bone marrow of the high-fat group, which contained increased proportions of T-lymphocytes with memory and activation markers, supported increased osteoclastic differentiation in coculture assay.

T-lymphocytes from the high-fat group, tested immediately ex vivo, show an increase in the message levels of several cytokines that have well-documented associations with inflammation and bone loss, including IL-6,(44) TNF-α,(45) and IL-1β.(46) The message levels for IFN-γ also were increased, but the role of IFN-γ in bone metabolism is somewhat controversial. One murine study showed that IFN-γ directly inhibits RANKL-mediated osteoclastogenesis by induction of TNF receptor associated factor-6 (TRAF6) degradation.(47) However, randomized, controlled trials have shown that IFN-γ did not prevent bone loss in patients with rheumatoid arthritis (RA).(48) Moreover, it was demonstrated recently that although IFN-γ can inhibit osteoclast formation directly; it also promotes bone resorption indirectly via antigenic-driven T-lymphocyte activation and subsequent secretion of RANKL and TNF-α so that the net effect of IFN-γ on bone remodeling was bone loss. The interaction between the immune and skeletal system is further strengthened by a recent study that showing that an imbalance of the RANKL/OPG ratio affected osteoblast activity with subsequent low bone formation in the adenosine deaminase (ADA)-deficient SCID mouse model.(49)

To our knowledge, our study provides the first demonstration of lipid-induced increased expression of the LOX-1 in T-lymphocytes in vivo. This receptor had been identified previously as the primary oxidized LDL receptor on endothelial cells,(16) and its expression on macrophages can be induced by TNF-α.(50) Interestingly, C-reactive protein (CRP), a nonspecific serum marker of inflammation that is a strong predictor of cardiovascular disease, hypertension, and diabetes(33,51) was shown recently to interact directly with LOX-1.(52) We had found previously that oxidized lipids induce LOX-1 expression by human T-lymphocytes in vitro.(38) Here we show that splenic T-lymphocytes from the high-fat group have higher LOX-1 expression, which theoretically could bind to circulating CRP, further enhancing T-lymphocyte activation and cytokine release. Ongoing studies in our laboratory are examining the potential role of CRP in the oxidized lipid induction of T-lymphocyte-mediated bone loss.

In addition to the enhanced inflammatory profile of splenic T-lymphocytes in the high-fat mice, several notable changes were observed in the BM as well. Indeed, other studies, not related to osteoporosis, have suggested that the BM can function as a secondary lymphoid organ, with mature T-lymphocytes migrating back and forth between blood and BM.(23,24) The BM also has been shown to play a central role in the maintenance of memory CD8 T-lymphocytes.(25) In this regard, it is highly relevant that the BM of the high-fat group contained significantly greater proportions of CD8 T-lymphocytes and activated memory T-lymphocytes and that this BM was able to support osteoclast formation in the complete absence of exogenous RANKL and macrophage colony-stimulating factor (M-CSF). A role for CD8 T-lymphocytes in bone metabolism had been documented previously in a study on postmenopausal women that showed that the proportion of CD8 T-lymphocytes coexpressing TNF-α and IFN-γ was significantly higher in the women with osteoporotic fractures than in the control subjects.(53) Consistent with that human study, our own results suggest that the BM cells from the high-fat diet mice may contain all the necessary factors to induce osteoclastogenesis.

An additional aspect of our study that is highly relevant to the human population is the fact that as early as 6 months of age the high-fat mice failed to show the usual increase in BMC (Fig. 1*B*). Studies aimed at elucidating the impact of body fat on bone density have suggested that subcutaneous fat is beneficial to peak bone mass, whereas visceral fat has negative effects on bone in both healthy and HIV^+^ individuals.(54–56) Moreover, Lac and colleagues showed that during the early growth period (ie, 35 days), rats fed a high-fat diet had lower bone BMC and BMD along with a negative correlation between visceral fat and BMD.(57) These findings are relevant to human health, where high-cholesterol diets are becoming more prevalent even in children, and evidence suggests that hyperlipidemic youth are more likely to be diagnosed with cardiovascular disease when they reach adulthood.(58) Furthermore, based on our own findings and reports by others, oxidized lipids not only can induce osteoclastic differentiation(22) but also can inhibit the differentiation of osteoblasts.(59) Moreover, Hirasawa and colleagues showed that 5-week-old *ApoE*^−/−^ mice (the same background strain as in this study) given a high-fat diet for 12 weeks had a greater number of osteoclasts in the trabecular bone, as well as increased osteoclast activity in the cortical endosteum, compared with control mice.(60) Therefore, hyperlipidemia and the subsequent effects of increased oxidized lipid levels not only may affect bone loss during aging but also may interfere with proper bone development early in life.

Based on our findings, we propose that T-lymphocytes may be one of the missing links in the documented lipid-mediated bone loss. There is increasing evidence that nutritional interventions may modulate oxidative stress, which can affect the generation of oxidized lipids. For example, the antioxidant cocoa polyphenol was reported to increase HDL, decrease LDL, and also decrease the oxidant susceptibility of LDL in both human and rodents.(61) Treatment with vitamin E has been shown to reduce inflammation-induced cytokine production in vitro not only by immune cells(62) but also by skeletal and cardiac muscle.(63) Furthermore, changes to diet and/or lifestyle that can decrease excess amount of visceral fat also can be beneficial. The results of this study suggest that these treatments may function, at least in part, by reducing the T-lymphocyte exposure to oxidized lipids.
